# Performing High-Quality Sublobar Resections: Key Differences Between Wedge Resection and Segmentectomy

**DOI:** 10.3390/cancers16233981

**Published:** 2024-11-27

**Authors:** Benjamin Bottet, Niek Hugen, Matthieu Sarsam, Mathias Couralet, Sonia Aguir, Jean-Marc Baste

**Affiliations:** 1 Department of General and Thoracic Surgery, Hospital Center University De Rouen, 1 Rue de Germont, F-76000 Rouen, France; benjamin.bottet@chu-rouen.fr (B.B.); matthieu.sarsam@chu-rouen.fr (M.S.); sonia.aguir@chu-rouen.fr (S.A.); 2 Netherlands Cancer Institute, Rijnstate Hospital, Amsterdam 1066CX, The Netherlands; nhugen@rijnstate.nl

**Keywords:** lung cancer, lung surgery, minimally invasive surgery, sublobar resection, lobectomy, video-assisted thoracoscopic surgery, robotic-assisted thoracoscopic surgery, precision surgery

## Abstract

Lung cancer remains the leading cause of cancer-related deaths worldwide. The introduction of targeted treatments and immunotherapy in lung cancer has transformed patient care by offering “precision medicine” focused on the characteristics of the disease. The same concept has emerged in lung cancer surgery. This “precision surgery” aims to determine the best surgical approach based on the characteristics of the cancer, the patient’s cardiorespiratory status, and technological advances in thoracic surgery. While lobectomy continues to be the gold standard for managing early-stage lung cancer, the implementation of screening programs has enabled the earlier detection of smaller tumors. In this context, sublobar resections, particularly segmentectomy, have emerged as valuable options in the treatment of early-stage lung cancer. Recent studies suggest that sublobar resections, including segmentectomy and wedge resection, provide a viable alternative to lobectomy. This review explores the various resection strategies available, tailored to patient and tumor characteristics, and highlights modern preoperative techniques aimed at advancing precision surgery.

## Simple Summary

This review explores the evolving landscape of lung cancer surgery, particularly focusing on the effectiveness and precision of sublobar resections such as segmentectomy. With advancements in screening and surgical techniques, there is a growing interest in more conservative approaches that spare lung tissue while ensuring complete cancer removal. The findings discussed in this review highlight the benefits and potential of sublobar resections, especially for early-stage non-small cell lung cancer, offering a less invasive option with comparable outcomes to lobectomy. This information is essential for the thoracic surgery community as they refine patient-specific treatment strategies in the era of precision medicine.

## 1. Introduction

Lung cancer remains one of the most frequently diagnosed cancers globally and is the leading cause of cancer-related deaths, accounting for a large proportion of cancer mortality [1].

The landscape of lung cancer diagnosis and treatment has evolved considerably in recent years. The introduction of lung cancer screening programs, such as low-dose CT scans, has played a pivotal role in enabling the earlier detection of lung cancer, particularly for conditions like ground-glass opacities. Early detection has significantly contributed to reduced mortality rates, allowing for timely interventions and more effective treatment strategies [2,3,4]. The advancement in screening programs has induced a significant evolution in the landscape of lung resection strategies. Notably, there has been a shift towards more precise and lung-sparing surgeries such as segmentectomy [5,6].

Minimally invasive techniques, including video-assisted thoracoscopic surgery (VATS) and robotic-assisted thoracoscopic surgery (RATS), have further revolutionized the field by offering enhanced precision and reduced trauma. These techniques utilize small incisions, avoiding the need for rib spreading, and have been shown to result in superior patient outcomes, including shorter hospital stays, reduced postoperative pain, and quicker recovery times compared to traditional open surgery [7,8,9,10,11,12,13,14].

Concurrently, the Enhanced Recovery After Thoracic Surgery (ERATS) program [15] and preoperative rehabilitation programs [16] have yielded a synergistic benefit. These measures collectively prepare patients on multiple fronts, optimizing their physical and physiological status for the upcoming lung surgery. Consequently, this personalized approach, enables patients who were considered marginal candidates in the past to undergo surgery nowadays.

The introduction of targeted treatments and immunotherapy in non-small cell lung cancer (NSCLC) has also transformed the management of lung cancer. Immunotherapy, which was initially introduced for metastatic or locally advanced NSCLC [17], has significantly enhanced patient survival rates [18] and is now also being considered for use in both neoadjuvant and adjuvant settings [19,20,21]. These new treatment modalities have transformed patient care by offering “precision medicine” focused on the clinicopathological characteristics of the disease [22]. More recently, the same concept has emerged in lung cancer surgery. This “precision surgery” aims to determine the best surgical approach based on the clinicopathological characteristics of the cancer, the patient’s cardiorespiratory status and technological advances in thoracic surgery [23].

The aim of this review is to explore the role of precision and customized surgery in the management of lung cancer, focusing on the current and future potential of sublobar resections in the context of early detection and individualized patient care.

## 2. Where Do We Stand?

Lobectomy has traditionally been regarded as the standard surgical approach for treating lung cancer [24]. However, with the advent of CT-based lung cancer screening, we have entered an era where “very early” NSCLC, such as tumors classified as T1a–bN0 (≤2 cm in size, peripheral, and node-negative), can now be detected at much earlier stages. This shift has opened the door to more conservative surgical options, including sublobar resections like segmentectomy or wedge. Recent guidelines and recommendations highlight the potential advantages of these procedures. While segmentectomy involves removing less lung tissue, it still aims to achieve complete tumor resection with sufficient margins and an adequate lymph node dissection to ensure proper staging and long-term oncological outcomes.

### 2.1. Segmentectomy as an Alternative: Early Adoption and Evolution Beyond High-Risk Patients

Initially, segmentectomy was proposed as a surgical option for patients deemed to be at a high risk of experiencing postoperative complications, particularly those with limited pulmonary reserve or significant comorbidities. A recent meta-analysis showed that segmentectomy demonstrated comparable survival outcomes and recurrence patterns when compared to lobectomy in elderly patient [25]. Anatomical segmentectomy, when combined with a thorough radical lymphadenectomy, emerges as a strong alternative for elderly patients, offering a balance between oncological efficacy and reduced surgical trauma [25,26]. Brunelli et al. demonstrated the safety profile of segmentectomy in high-risk patients. Their analysis showed that in that patient cohort, the relative risks of cardiopulmonary morbidity and 30-day mortality were significantly lower for segmentectomy, with relative risk ratios of 0.71 and 0.65, respectively, compared to lobectomy [27]. These results underline the value of segmentectomy as a less invasive but effective surgical option for patients with early-stage lung cancer, particularly for those for whom lobectomy may present a higher risk.

Segmentectomy is not only indicated for patients with compromised pulmonary function or significant comorbidities [28,29] but also for carefully selected cases involving specific tumor characteristics. This procedure is particularly useful for patients with small, early-stage lung cancers, including pure ground-glass opacities measuring less than 2 cm, or minimally invasive or invasive adenocarcinomas measuring less than 2 cm [5,24,30]. Segmentectomy can be performed provided that sufficient resection margins, generally greater than 1 cm or equivalent to the size of the tumor, are ensured [31,32]. A recent meta-analysis by Winckelmans et al. confirmed that for tumors under 2 cm, segmentectomy provided oncological outcomes comparable to those of lobectomy, without sacrificing long-term survival or increasing recurrence rates [6]. Additionally, research by Suzuki et al. demonstrated that segmentectomy could maintain equivalent oncological control while minimizing pulmonary compromise [32]. According to Tane et al., patients who underwent thoracoscopic segmentectomy demonstrated significantly better postoperative lung function compared to those who had lobectomy. The study found that segmentectomy preserved approximately 91.9% of preoperative lung function, compared to 81.7% in the lobectomy group, highlighting its benefit for long-term respiratory outcomes [33]. Furthermore, 3D-CT imaging, used to plan segmentectomy, allows for an accurate assessment of lung volume and helps guide surgeons in preserving as much functional lung tissue as possible during the procedure.

These results underline the growing role of segmentectomy in thoracic oncology, particularly as a lung-sparing alternative in selected patients with NSCLC. By preserving more lung tissue, segmentectomy offers a crucial advantage in maintaining postoperative quality of life, particularly in patients with limited lung function or those who may require further surgery.

### 2.2. RATS vs. VATS: Minimally Invasive Approaches

When deciding between RATS and VATS for segmentectomy, several factors must be considered. Both techniques are minimally invasive and have demonstrated excellent outcomes in lung cancer surgeries, but there are key differences. RATS provides enhanced dexterity and three-dimensional visualization, making it especially useful for complex segmentectomies where precise dissection of segmental vessels and bronchial structures is required. According to Leung et al., there has been a significant increase in the use of robotic-assisted techniques for segmentectomies in early-stage NSCLC. This trend suggests that robotics may facilitate more segmentectomies for early-stage lung cancer, offering a less invasive, yet effective, surgical option compared to VATS [34]. Wu et al. showed that RATS could offer a slightly longer disease-free survival than VATS, while overall survival rates remained comparable [35].

RATS also offers a reduced risk of intraoperative complications, such as bleeding, and may result in a shorter hospital stay, particularly in elderly or high-risk patients [36]. Moreover, some studies have demonstrated that RATS leads to better perioperative outcomes, including reduced intraoperative bleeding and shorter hospital stays, in overweight and obese patients compared to VATS [37,38]. Ultimately, the choice between RATS and VATS should be based on the complexity of the surgery, the patient’s health status, and the surgeon’s expertise. Both methods provide viable options for lung-sparing surgeries like segmentectomy, with RATS potentially offering advantages in more intricate cases.

### 2.3. Recent Trials and Evidence Supporting Sublobar Resection for Early-Stage NSCLC

Recent research has led to a major shift in the surgical management of early-stage NSCLC. A key study, led by Saji et al., compared the outcomes of segmentectomy versus lobectomy for patients with small-sized peripheral NSCLC. After a median follow-up of 7.3 years, the results revealed no significant difference in overall survival between the two groups, although segmentectomy was associated with slightly lower relapse-free survival. These findings support the non-inferiority of segmentectomy as a viable surgical alternative for carefully selected patients with early-stage peripheral NSCLC [39].

Another study, the CALGB 140503 (Alliance) trial, reported by Altorki et al., further reinforced these findings. This multicenter, non-inferiority phase 3 study randomized 697 patients with peripheral stage IA NSCLC between lobectomy and sublobar resection (segmentectomy or wedge resection). After a median follow-up of 7 years, sublobar resection proved non-inferior to lobectomy in terms of disease-free survival and overall survival. Disease-free survival rates at 5 years were 63.6% for sublobar resection and 64.1% for lobectomy, with similar overall survival rates of 80.3% and 78.9%, respectively [40]. A post hoc analysis of CALGB 140503 [41] revealed no statistically significant differences in disease-free survival, overall survival, and lung cancer-specific survival among patients who underwent lobectomy, segmentectomy, and wedge resection. Regarding respiratory functions, the median reduction in forced expiratory volume in 1 s (FEV1) between the two groups was minimal, ranging from 2% to 3.5%. This marginal disparity in FEV1 reduction could prompt a reevaluation of the benefits of segmentectomy versus lobectomy, particularly in patients with compromised pulmonary function, where the impact of lobectomy may be more significant. As highlighted by Boujibar et al., this leads to the consideration of alternative, more pertinent and clinical relevant markers for evaluating respiratory capacity [42]. These studies provide vital information on the evolution of early-stage lung cancer management, expanding the surgical options available to patients. By proposing a more conservative approach with a sublobar resection, these studies highlight the potential benefits of preserving lung function without compromising oncological outcomes. The findings underscore the growing role of personalized, lung-sparing surgical techniques in the era of precision medicine and surgery [43].

### 2.4. Challenges in Lymph Node Decision and Recurrence Rates

The rate of loco-regional recurrence following segmentectomy has been reported to be notably higher compared to lobectomy [39,40]. This increased recurrence rate may be partly attributed to the lack of comprehensive lymph node dissection, especially in cases where a wedge resection is performed. Both Brunelli et al. and Saji et al. recommended the use of a frozen-section analysis on segmental lymph nodes during segmentectomy to enhance the thoroughness of the nodal evaluation [39,44]. Seitlinger et al. introduced the concept of sentinel lymph node detection in lung cancer, highlighting cases where lymphatic drainage extends to the mediastinum [45]. According to current recommendations, in tumors ≤3 cm located in the outer third of the lung with no lymph node enlargement on CT or uptake on PET-CT, direct surgical resection with systematic nodal dissection is indicated. However, for central tumors or cases with N1 nodes, preoperative mediastinal staging is advised [30,46].

Furthermore, Lin et al. demonstrated that the maximum standardized uptake value (SUVmax) of the primary tumor was an independent risk factor for occult N2 metastases, (OR = 0.88 (95% CI: 0.81–0.96; *p* = 0.003)) [47]. Similarly, Gao et al. addressed the unique challenges posed by solid tumors in early-stage NSCLC when using PET-CT for mediastinal staging. The authors noted that solid tumors larger than 2 cm or with high metabolic activity were more likely to have occult lymph node metastases, even in patients staged cN0 by PET-CT [48]. Adachi et al. showed in their retrospective cohort that patients with purely solid tumors and SUVmax > 2 were significantly more likely to develop occult N2 than those without these two factors (0.9% vs. 8.4%, *p* = 0.005). Similarly, the 5-year cumulative incidence of recurrence was significantly higher in these patients compared to those without these factors (15.9% vs. 2.0%, *p* < 0.001) [49]. Additionally, some studies, highlighted the notion that pure-solid nodules carry a higher risk of recurrence [50,51,52]. While segmentectomy and lobectomy demonstrated similar recurrence-free survival in patients with small pure-solid NSCLC, the rate of loco-regional recurrence was higher after segmentectomy. Subgroup analyses revealed that male patients and those older than 70 years had better overall survival with segmentectomy compared to lobectomy, further highlighting the importance of careful patient selection [50]. Motono et al. proposed that sublobar resection could be a viable option for patients with a consolidation-to-tumor ratio (CTR) of 0.5 or lower, offering the benefit of reducing the risk of postoperative complications [53].

### 2.5. Management of Intersegmental Plane

The identification and management of the intersegmental plane are key components in performing a successful lung segmentectomy, as they ensure precise tumor resection while preserving healthy lung parenchyma. Several techniques are employed to visualize and separate this plane. One traditional method is the inflation–deflation technique. This creates a natural visual boundary between the segments, making it easier to identify the plan. However, this method may prove less reliable in patients with significant collateral ventilation, making identification of the resection line difficult [54]. A more modern and increasingly popular technique is the use of indocyanine green fluorescence imaging [55]. After sectioning the arteries destined for the segment, indocyanine green is injected, enabling the surgeon to visualize the segments still vascularized, thanks to the fluorescence [54,56,57,58].

When it comes to separating the intersegmental plane, surgeons typically choose between staplers and energy devices. Staplers are often preferred because they reduce the risk of air leaks and bleeding, ensuring faster recovery compared to energy dissection [59].

## 3. The Different Types of Sublobar Resections

Sublobar resection represents a highly valuable option for treatable metastatic lesions. In the case of a controlled disease with pulmonary oligoprogression, sublobar resection emerges as a strategic choice, as it not only effectively addresses the localized progression but also emphasizes the importance of preserving lung parenchyma [60].

Segmentectomy is indicated not only for patients with poor lung function but also for specific cases and selected patients with a pure ground-glass opacity tumor of <2 cm, or a NSCLC of <2 cm (cT1a-bN0), if expected margins are >1 cm or measuring at least the size of the tumor [6]. Two recent articles (CALGB 140503 trial [41] and JCOG0802 trial [39]) have presented evidence that in cases of early-stage NSCLC, the consideration of segmentectomy or wedge resection is viable, provided certain critical principles are meticulously adhered to. These principles emphasize the necessity of achieving adequate resection margins and confirming the absence of lymph node involvement.

Before considering sublobar resection, a thoughtful evaluation of critical factors and precise questioning is imperative, including the following key considerations.

### 3.1. Patient Profile

Is the patient young or elderly? Does he have any frailties or comorbidities? Does the patient have impaired pulmonary function? Performance status score?

Age plays a crucial role in determining the appropriate surgical approach for lung cancer resection, particularly for early-stage NSCLC. Several studies suggest that older patients, especially those with additional comorbidities, benefit more from sublobar resections rather than lobectomy with a secure surgical margin [29,50,61,62].

Benker et al. demonstrated that advanced age, a low BMI, and a reduced FEV1 were significant predictors of increased complication risk and shorter long-term survival in patients undergoing NSCLC resection. The study highlighted that cardiac complications were more commonly associated with hypertension and coronary artery disease, while pulmonary complications were linked to a high pack-year smoking history [63]. Chronic obstructive pulmonary disease was related to the increased risk of death within 5 years after surgery [62]. Similarly, Rosen et al. found that increasing age, male sex, the presence of comorbidities, and decreased facility volume were all associated with higher 30-day mortality rates and extended lengths of stay [64]. Furthermore, Huang et al. found that patients with diabetes had significantly higher 30- and 90-day mortality rates compared to non-diabetic patients (*p* < 0.001), largely due to the presence of higher preoperative comorbidities [65].

The assessment of impaired pulmonary function in patients undergoing lung resection is typically guided by established preoperative evaluations (forced expiratory volume in one second and diffusing capacity for carbon monoxide). A predicted postoperative FEV1 or DLCO below 60% requires further evaluation due to an increased risk of perioperative complications. Values below 30% are considered high risk for major lung resections. Additionally, the guidelines recommend the use of exercise testing, such as cardiopulmonary exercise testing, which measures VO2max. A VO2max below 10 mL/kg/min indicates a significantly increased risk of postoperative complications and mortality [66,67]. In cases where VO2max is not available, low-tech exercise tests (e.g., stair climbing and the 6MWT) provide valuable information about a patient’s functional capacity [68,69].

### 3.2. Nodule Characteristics

Is it a peripheral or palpable lesion, or a central or ground-glass opacity (CTR)? Is the histology based on a preoperative biopsy known?

According to Motono et al., lesions with a CTR of 0.5 or lower may be ideal candidates for sublobar resections, as they typically have lower malignancy potential and are less invasive. Additionally, recent findings from a multicenter phase 3 trial in Japan support the use of segmentectomy as a standard treatment for NSCLC up to 3 cm in size, particularly for tumors with ground-glass opacity (GGO) or predominant GGO characteristics, achieving an impressive 5-year relapse-free survival rate of 98.0% [70]. Solid nodules, on the other hand, particularly those with a high SUVmax or located centrally, present a higher risk of occult metastasis, and may require a more aggressive surgical approach [47,49,50,51]. Maeda et al. showed, in stage IA, the 5-year recurrence-free probability for adenocarcinoma was significantly higher than that for squamous cell carcinoma (SCC) (91.4% and 82.6%, respectively). No difference was observed in stage IB. On the contrary, in stage II, the 5-year recurrence-free probability for adenocarcinoma was significantly lower than that for SCC. The overall survival rate was higher for adenocarcinoma compared to SCC [71].

Where is the lesion located in the segment and how far is it from the intersegmental plane? What is the resection margin? Are there anatomical variations? Is there any predictable difficulty in dissection?

The use of 3D reconstruction has transformed the preoperative planning process for sublobar resections. It allows for the precise visualization of the tumor in relation to the intersegmental plane, ensuring that surgeons can achieve adequate resection margins, even in anatomically challenging areas [72,73]. Guidelines for non-small-cell lung cancer recommend a resection margin of at least 2 cm, or a margin equivalent to the size of the tumor, for sublobar resections [74]. Indeed, some authors have shown that an adequate margin is associated with a significant reduction in local recurrence rates [75,76]. However, opinions differ in the literature as to the extent to which the distance of the resection margin influences local recurrence [77,78]. Okubo et al. demonstrated that achieving larger margins in complex segmentectomies was more challenging but crucial, especially for tumors located in difficult areas [58]. Bertolacinni et al. showed in their meta-analysis that simple and complex segmentectomies had comparable postoperative outcomes: similar postoperative complication rates and length of hospital stay [79]. Some studies highlighted that complex segmentectomies were associated with a longer operative time [79,80].

What are the implications of Spread Through Air Spaces (STAS) in determining the extent of surgical resection?

STAS was first defined in 2015 as the spread of cancer cells, in the form of small clusters, into the air spaces of the lung parenchyma beyond the main edge of the tumor [81]. This mode of invasion is associated with poorer outcomes in NSCLC [82]. According to Li et al., patients with stage I NSCLC with STAS had significantly lower recurrence-free survival and overall survival compared to those without STAS, particularly when undergoing sublobar resection [83]. Some authors corroborated these findings, showing that segmentectomy offered a recurrence-free survival rate comparable to lobectomy in patients with STAS, while wedge resection was associated with a higher risk of recurrence in this patient population [84,85]. Additionally, Jung et al. demonstrated that STAS was an independent risk factor for recurrence in lung adenocarcinoma patients at stages pT1b and pT1c, but not at stage pT2a [86]. The feasibility of detecting STAS intraoperatively through a frozen-section analysis has been explored, with some studies showing good specificity but limited sensitivity [87,88]. As Mino-Kenudson et al. highlights, processing artifacts frequently encountered during frozen-section preparation can compromise the accuracy of STAS identification. Additionally, evaluating invasive components, STAS, and histologic subtypes through a frozen section is time-consuming, potentially leading to significant delays in surgery. The assessment also suffers from high interobserver variability, reducing its overall accuracy and applicability as a consistent intraoperative diagnostic tool [89].

**Figure 1:**
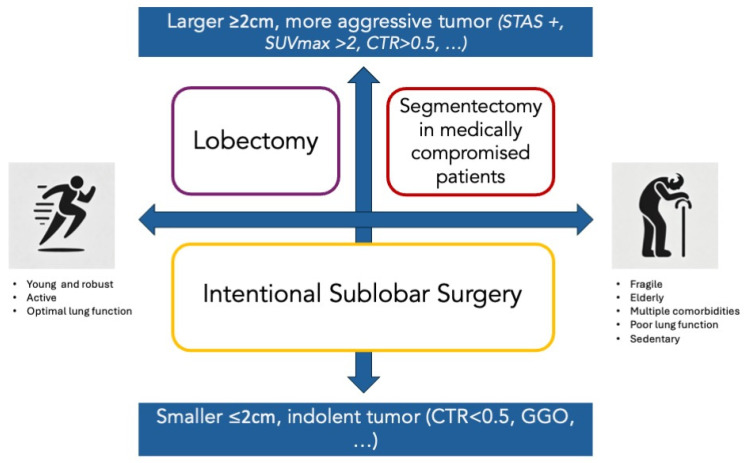
Decision-making flowchart for surgical approach in lung cancer resection. This figure illustrates the decision-making process for surgical treatment of early-stage lung cancer. The purple upper left panel represents the selection of lobectomy for fit patients who can undergo this procedure, as recommended by oncological guidelines. The red panel in the upper right corner addresses decision-making for medically compromised patients affected by frailty, age, comorbidities, or reduced pulmonary function. For these patients, sublobar surgery may be recommended to minimize the impact of the procedure, such as preserving postoperative pulmonary function, while accepting a potentially higher risk of oncological recurrence. However, if tumor characteristics allow, these patients may also qualify for intentional sublobar surgery, similar to fit patients (yellow panel). STAS: spread through air spaces, SUVmax: maximum standardized uptake value, CTR: consolidation-to-tumor ratio, GGO: ground-glass opacity.

Thanks to this comprehensive assessment, four distinct scenarios can be established, each guiding the choice of an appropriate surgical strategy. These scenarios allow for precise decision-making and tailored patient care (Graphical abstract, Figure 1).

### 3.3. Wedge Resection

**Figure 2:**
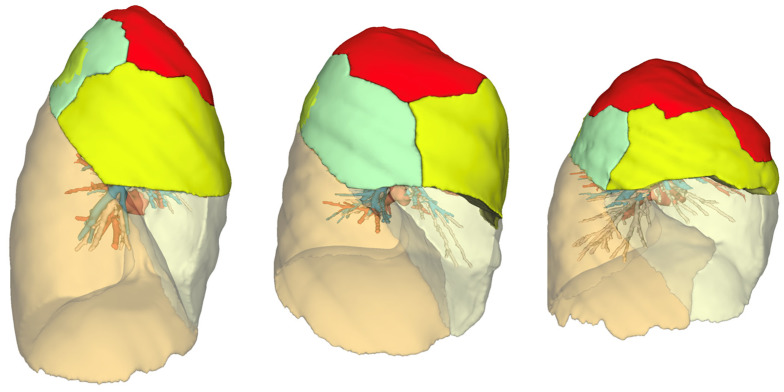
Three-dimensional reconstructions of the upper lobe: segmental shape and volume variations 3D models generated using the Visible Patient^®^ software V 1.0.15, demonstrating the different shapes and volumes of the segments in the upper lobe. Each segment is highlighted in a distinct color to illustrate anatomical variations (S1 in red, S2 in green, and S3 in yellow).

Wedge resection is an optimal approach for treating slowly progressive peripheral lesions characterized by low PET scan uptake and as a diagnostic procedure. This surgical option is particularly well-suited for patients who may be considered vulnerable due to numerous comorbidities or advanced age. The primary objective of this procedure is to ensure rapid surgery, typically within 30 min, with sufficient resection margins, while minimizing the associated morbidity. Furthermore, in select cases, surgeons may opt to conduct hilar or mediastinal lymph node dissection (Figure 2).

### 3.4. Atypical Segmentectomy

An atypical segmentectomy or a semi-anatomical segmentectomy [90] involves the dissection of the pulmonary hilum, with the potential need to divide a vascular or bronchial component to facilitate the release of the lung parenchyma for stapling. This surgical technique is recommended for less peripheral lesions where the assurance of adequate margins is challenging to achieve through a wedge resection. This procedure is preferred in frail patients or “compromised patients” or those with lung metastases. Hilar lymphadenectomy is performed. It is distinguished by its short operative time and lower associated morbidity, mainly due to the reduced need for extensive dissection of bronchovascular structures compared with typical segmentectomy (Figure 2).

### 3.5. Typical Segmentectomy

A typical segmentectomy is defined as an anatomic lung resection smaller than a lobectomy and including the dissection and division of the corresponding segmental artery/arteries, bronchi, and veins. In some cases, segments may not require the individual division of the segmental vein as venous tributaries are divided along the intersegmental plane [44]. This procedure ensures good resection margins and complete lymph node dissection. Dissection of bronchovascular structures is more time-consuming than with atypical segmentectomy (Figure 2).

### 3.6. Extended Segmentectomy

When the lesion is situated in proximity to an intersegmental plane, an extended segmentectomy offers a valuable approach to secure adequate resection margins. In extended segmentectomy, the resection line can extend beyond traditional anatomical segments, by adding only peripheral lung resection without additional higher resection.

Similar to typical segmentectomy, this procedure ensures good resection margins and allows lymph node dissection. Unlike a bi-segmentectomy, extended segmentectomy involves less extensive manipulation of bronchovascular structures, thereby reducing the risk of injury or errors [91].

## 4. The Essential Tools for Performing a Sublobar Resection

### 4.1. Contrast-Enhanced Thoracic CT Scan

Accurate preoperative radiologic evaluation of the anatomic relations and features of pulmonary tumors is essential to determine the appropriateness of surgical resection and the type of resection (anatomic vs. nonanatomic) required. A thoracic CT scan is essential for surgical planning. Stefanidis et al. described a comprehensive template for preoperative radiologic analysis of the lung [92]. However, preoperative CT does not always accurately predict the completeness of the pulmonary fissures.

The pulmonary vessels have a wide range of anatomic variations. These variations can lead to incidental vessel injuries, potentially necessitating more extensive lung resections if not properly identified and repaired [93]. Identifying these anatomic variants before surgery and addressing them carefully during the procedure enhances the accuracy and safety of lung resections. Many of these variants can be identified and reported by the radiologist following a contrast-enhanced thoracic CT. Some bronchial anomalies can cause failure during general anesthesia and selective single-lung ventilation, leading to unintended ventilation of the operated lung. Preoperative identification of these anomalies allows for adjustments in the single-lung ventilation strategy. Contrast-enhanced thoracic CT is a rapid and readily available tool for assessing the feasibility of sublobar resection.

### 4.2. Three-Dimensional Reconstruction

The increasing interest in segmentectomy, which is regarded as a more complex surgical procedure than lobectomy, has introduced unique challenges that were rarely encountered in traditional open surgery [91,94]. These challenges often stem from anatomical variations in bronchovascular structures, which are more frequently encountered in segmentectomies than in lobectomies [44,95].

Three-dimensional reconstructions have become essential in the planning of pulmonary segmentectomies due to significant interindividual variation in segment volumes and bronchovascular structures (Figure 1). It plays a pivotal role in the safety and precision of anatomical segments. These reconstructions not only allow for the precise localization of the target nodule but also play a critical role in ensuring adequate resection margins. The incorporation of safety margins into the surgical strategy is facilitated by 3D models. This crucial step reinforces the oncological robustness of the planned resection, guaranteeing complete removal of the tumor while minimizing the risk of residual disease [72,73].

By defining the segmental vascularization and segmental bronchial tree division, it could identify possible anatomical abnormalities that could make the intervention complex or impossible.

Three-dimensional reconstructions may aid the surgeon in the preoperative phase to consider segmentectomy in favor of lobectomy in a patient with compromised lung function. In regard of personalized medicine, this may present surgery as a valuable alternative to external beam or stereotactic radiotherapy in frail patients.

By providing a detailed description of the lung segments (nodule position, intersegmental plane, vascular and bronchial anatomy), the surgeon can draw up a precise preoperative surgical strategy, also called preoperative planification or simulation, and determine the type of sublobar resection [55,96].

To mitigate these challenges, ensure compliance with oncological surgical principles, and prioritize patient safety, the European Society of Thoracic Surgeons (ESTS) has developed a set of recommendations for the performance of segmentectomies [44].

### 4.3. Preoperative Marking of Nodules

An increasing number of patients are now being referred to surgeons due to suspected pT1a NSCLC or ground glass opacities (GGO). One notable characteristic of these newly discovered lesions lies in their challenging detection, primarily owing to their small size and the inconsistency in their palpability, especially in the case of GGO.

Preoperative planning plays a pivotal role in determining the surgical approach and the type of resection required [96]. In the era of sub-lobar resection, where precision is essential, the utilization of preoperative lesion tracking techniques has become indispensable in certain surgical scenarios [55,97].

To overcome this difficulty, various percutaneous CT-guided techniques have been developed to localize small lung nodules, such as percutaneous hooks, coils, or radioactive iodine seeds [98]. Because these percutaneous techniques are often performed in radiology departments located far from the operating room (OR), good coordination and logistics are mandatory between radiologists, anesthesiologists, and thoracic surgeons. These procedures can lead to complications such as pneumothorax, bleeding, and migration of the hooks or coils [73,99]. These potential complications could be reduced by performing these techniques immediately before surgery in the OR, thanks to hybrid rooms equipped with C-arm CT [100] or electromagnetic navigation bronchoscopy (ENB) [101]. A notable disadvantage of these sophisticated devices is the substantial costs associated with their installation, maintenance, and staff training, which can be out of reach for some healthcare facilities. The ideal method for localizing pulmonary nodules before surgical resection should meet criteria of accuracy, simplicity, and safety, ideally performed within the same operative setting as the resection itself. Various bronchoscopy techniques have been documented for this purpose. Virtual bronchoscopy and Radial Endobronchial Ultrasound (EBUS) [99] could offer advantages in terms of cost-effectiveness, and reproducibility. This technique enables the nodule to be located and the visceral pleura to be marked by injection of methylene blue [99], combined with ^99m^Technetium [102], or indocyanine green [103].

**Figure 3:**
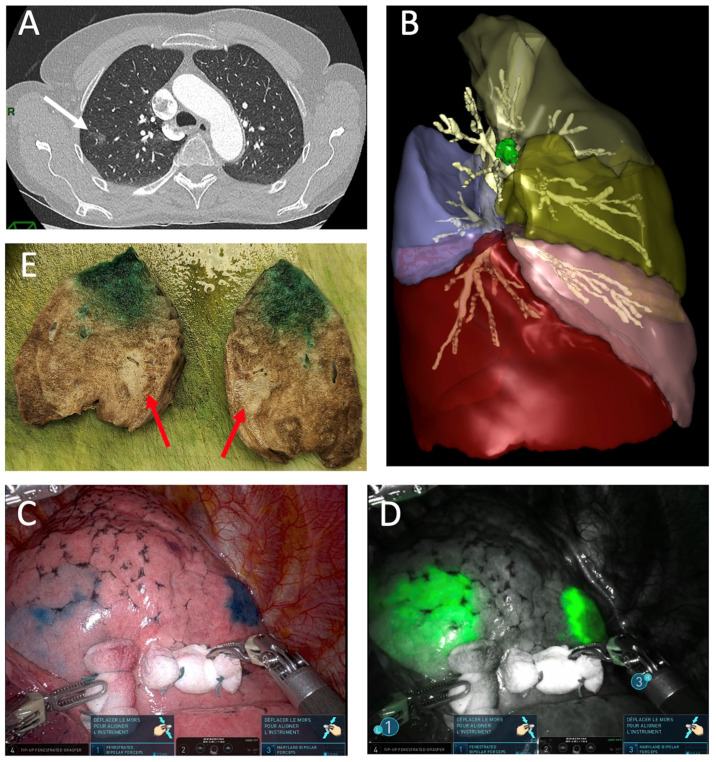
Multimodal robotic approach for a ground-glass opacity lesion in the right upper lobe. (**A**) Chest CT scan showing a ground-glass opacity lesion in the right upper lobe (white arrow); (**B**) 3D reconstruction using Visible Patient^®^, displaying the lesion in S2, near S1 and S3 segments; (**C**,**D**) robotic surgical approach with methylene blue and indocyanine green dye marking the lesion anteriorly and posteriorly; (**E**) pathological specimen with the tumor indicated by red arrows.

These techniques have become essential tools in the arsenal of thoracic surgeons, ensuring that patients receive the highest standard of care while benefiting from the advantages of minimally invasive surgery (Figure 3).

## 5. Conclusions

In the current era of precision medicine in oncology there is an ongoing search for tumor and patient characteristics that may customize patient care. A deeper understanding of the molecular profile and targetable driver mutations is sought. In lung cancer, this approach has significantly impacted patient care thanks to targeted therapies and immunotherapy. In parallel with the principles of precision medicine, the concept of precision surgery has been integrated into the field of surgical oncology. The primary objective is to provide patients with tailored surgical interventions, carefully considering their overall health, age, comorbidities, disease characteristics, lung anatomy, and lesion location. The introduction of 3D reconstructions has revolutionized our approach to lung cancer surgery, allowing for the optimization of surgical plans and prioritizing patient-centered care.

This personalized approach enhances the precision and safety of segmentectomy procedures, ultimately leading to improved outcomes and minimized postoperative complications.
